# Severe Rhabdomyolysis Associated with Acute Amphetamine Toxicosis in a Dog

**DOI:** 10.1155/2020/2816373

**Published:** 2020-01-29

**Authors:** M. Ryan Smith, Virginie A. Wurlod

**Affiliations:** Department of Veterinary Clinical Sciences, Louisiana State University, Skip Bertman Drive, Baton Rouge, LA 70803, USA

## Abstract

A 3-year-old female spayed rat terrier presented for hyperactivity and repetitive circling to the right of less than one-hour duration. On examination, the patient was dehydrated, hyperactive, and dysphoric. Laboratory tests initially revealed elevations in creatine kinase (CK) and aspartate aminotransferase (AST). Serial chemistries indicated significant progression of CK elevation to a maximum of 181,900 U/L on day 3 along with the development of profuse myoglobinuria. A urine drug screening test was positive for amphetamine metabolites. This patient was treated with sedatives, aggressive fluid diuresis, and antioxidants. The dog recovered uneventfully with no indicators of renal dysfunction based on serial blood chemistries and was discharged five days after presentation. Follow-up blood chemistries taken four days after discharge revealed near normalization of CK and resolution of myoglobinuria. This case report describes a particularly severe case of rhabdomyolysis associated with amphetamine toxicity and its successful treatment.

## 1. Introduction

Amphetamine exposure has become increasingly reported in veterinary species due largely to the prevalence of its use in humans, both medically and recreationally [1–3]. Toxicosis occurs because of the drugs' sympathomimetic activity derived from the release of catecholamines, primarily dopamine and norepinephrine, and the reduction of their clearance [2, 4, 5]. The stimulant activity of amphetamines results in increased muscle activity and, thus, increased energy demand. As cellular energy is depleted, the functionality of calcium ion pumps is hindered leading to increased calcium levels in the sarcoplasmic reticulum. The result of this homeostatic breakdown is prolongation of muscle contractions and worsening of fasciculations which further exacerbates energy demands. Activation of calcium-dependent proteases and phospholipase occurs and causes physical degradation of the cellular structure resulting in myolysis [3, 6–8]. Additionally, hyperthermia may develop due to the excessive muscular activity, which can damage myocytes directly and further exacerbate metabolic demands [9]. Rhabdomyolysis is a frequently reported complication associated with amphetamine toxicity in humans, occurring in up to 42% of cases [3, 6, 7]. The resultant myoglobinuria has been linked to acute kidney injury [6–8]. Amphetamine-associated rhabdomyolysis is noted to occur in the veterinary literature, but reports are uncommon and ill-defined [4, 10, 11]. This report describes a severe case of amphetamine-associated rhabdomyolysis in a canine patient without development of acute kidney injury using only supportive therapies.

## 2. Case Presentation

A 3-year-old female spayed rat terrier presented to the Louisiana State University emergency service for hyperactivity and circling to the right. The clinical signs were first noted less than one hour prior to presentation shortly after attending an adolescent's birthday party. On presentation, the patient weighed 6.8 kg and was dehydrated (5-7%), hyperthermic (39.4°C), and tachycardic (216 beats per minute). Pertinent physical examination findings included bilateral mydriasis with minimally responsive pupillary light reflex, generalized ataxia, incessant circling to the right, and mentation changes consistent with dysphoria. Point-of-care assessments indicated hemoconcentration (PCV 61% and TS 82 g/L), hypoglycemia (3.2 mmol/L), and hyperlactatemia (3.0 mmol/L). An initial systolic blood pressure taken via Doppler flow was 152 mmHg. Electrocardiogram (ECG) evaluation revealed sinus tachycardia with intermittent ventricular premature contractions.

Initial supportive therapy consisted of a 10 mL/kg bolus of intravenous (IV) lactated Ringers solution (LRS) followed by a rate of 5 mL/kg/hr. The patient received a bolus of 25% dextrose (1 mL/kg) due to hypoglycemia and was subsequently supplemented with dextrose added to LRS at 5%, then titrated to need.

Initial serum chemistries indicated significant elevations in creatine kinase (1,481 U/L; reference range: 0–200 U/L) and total bilirubin (41 *μ*mol/L; reference range: 0–6.8 *μ*mol/L). Additional alterations included mild elevations in aspartate aminotransferase (AST), sodium, and chloride as well as mild hypocalcemia and hypophosphatemia (Table 1). Acute toxicosis was suspected based upon the history and initial findings. Toxicant differentials included stimulants such as methylxanthines, cocaine, amphetamine derivatives, or serotonin syndrome as seen with serotonin reuptake inhibitors.

Sedative treatment was then started due to the degree of hyperactivity displayed by the patient. Acepromazine was administered to a total dose of 0.04 mg/kg IV but was ineffective. A single dose of midazolam was then administered at a dose of 0.25 mg/kg IV; however, the patient's hyperactivity became exacerbated. Additional sedative treatments attempted included butorphanol at 0.5 mg/kg IV and dexmedetomidine at 3 *μ*g/kg IV, each to marginal effect. Cyproheptadine was initiated at 1.2 mg/kg PO to treat for the possibility of serotonin syndrome, after which the patient's hyperactivity improved. Vital parameters, blood pressure, and blood glucose were monitored, and continuous ECG telemetry was maintained overnight.

The following day, significant pigmenturia of a dark brown color was noted. The presence of pigmenturia persisted in spite of centrifugation. The differentials for the patient's pigmenturia included myoglobinuria, bilirubinuria, and hemoglobinuria. Subsequent blood chemistry evaluation (day 2) indicated progressive elevation of CK (160,610 U/L), ALT (342 U/L), and AST (4,251 U/L) suspected to be the result of severe rhabdomyolysis with or without concurrent hepatic injury (Table 1). Marginal hemolysis was noted on the blood sample. Based upon the characteristics of the pigmenturia and current blood chemistry parameters, myoglobinuria secondary to rhabdomyolysis was considered likely.

**Figure 1:**
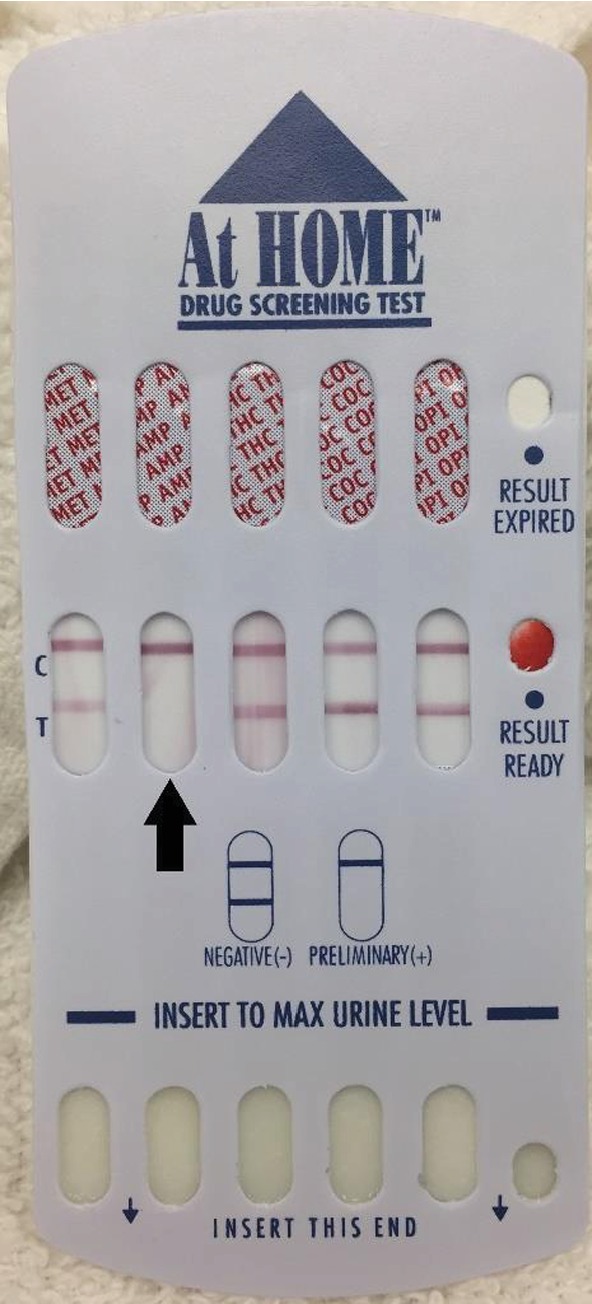
Drug screening test card utilized for this patient. Note the absence of a testing line, denoted as “T” on the test card, on the amphetamine test strip indicating a positive result (black arrow).

Treatment was altered by discontinuing all sedatives with exception to acepromazine, of which the dose was increased to 0.25 mg/kg IV as needed for effective control of hyperactivity. Cyproheptadine was continued at the previous dose, and N-acetylcysteine was added at 70 mg/kg IV for hepatic support and antioxidant properties. Fluids were maintained at the previous rate to promote diuresis due to the risk of acute kidney injury associated with severe myoglobinuria. A urine sample obtained at presentation was then screened for illicit substances using an At Home Drug Screening Test (Phamatech Laboratories & Diagnostics, San Diego, California, USA). Testing displayed a positive result for amphetamines (Figure 1). Based on this result and a clinical picture consistent with the disease, a diagnosis of amphetamine toxicosis was considered likely. Confirmatory testing by gas chromatography/mass spectrometry (GC/MS) (Texas Veterinary Medical Diagnostic Laboratory) was submitted using a fresh sample approximately 72 hours after admission. Unfortunately, the testing was unable to yield a confirmatory result for amphetamines.

Serum chemistries were evaluated again on the third and fifth days of treatment to track muscle enzyme activity and to monitor for development of azotemia (Table 1). Further elevation of CK (181,900 U/L) was noted on the third day but was significantly improved by the fifth day (4,410 U/L). All clinical signs had resolved by the fourth day of treatment, and the patient was discharged five days after admission with oral S-adenosylmethionine (Denamarin; Nutramax Laboratories, Lancaster, South Carolina, USA) for continued antioxidant support and amoxicillin/clavulanic acid (Clavamox; Zoetis, Parsippany, New Jersey, USA) for a catheter site infection. Reevaluation of serum chemistries four days after discharge (day 9 after admission) indicated near normalization of all parameters (Table 1) with the patient having developed no observable complications attributable to rhabdomyolysis.

## 3. Discussion

This case report describes the development of severe rhabdomyolysis attributed to amphetamine toxicity in a dog. Rhabdomyolysis as a sequela of amphetamine toxicity has been rarely reported in veterinary cases; however, the severity of the associated rhabdomyolysis seen in this patient has not been previously described [10, 11].

The diagnosis of amphetamine toxicity in this case was based upon a positive result on a urine drug screening test along with clinical signs and laboratory findings consistent with published reports of amphetamine toxicity [4, 10–12]. Urine screening tests for illicit drugs have been found to be effective in dogs exposed to amphetamines [13]. False positives can occur, usually caused by interfering substances and cross-reactivity from other medications [14]. The patient in this report was not exposed to any of the known cross-reactive substances at the time of initial sampling; thus, we considered this patient's result to be valid. Confirmatory testing by GC/MS was attempted, but it did not yield a positive result. The submission of a urine sample taken roughly 72 hours after admission was required because the remaining volume of urine from the initial sampling was inadequate for test submission. The length of time that amphetamine can reliably be detected in urine is approximately 48 hours in humans, though urinary elimination of amphetamine in dogs is thought to occur in as little as 6 hours [4, 14]. It is considered likely that the sample submitted for GC/MS testing fell outside of the time for detection, thus yielded a negative result.

Laboratory derangements reported in amphetamine toxicity in the veterinary literature commonly cite the potential for rhabdomyolysis, but it is only sporadically reported [10, 11]. In this patient, the most likely explanation is that dramatically increased muscle activity caused a metabolic demand that could not be met, ultimately resulting in disruption of cellular homeostatic mechanisms and myocyte degradation [3, 6, 9]. Hyperthermia is commonly associated with amphetamine toxicity, and heat-related myocyte injury has been described in veterinary medicine [15]. This mechanism, however, is considered unlikely in this case because the reported temperature at which heat-related myocardial injury occurs (42°C) is higher than the maximum measured temperature in this patient (39.9°C) [9]. Electrolyte derangements relating to calcium and potassium are reported as commonplace in patients with rhabdomyolysis, but they did not appear to be a factor in this patient [8]. It is possible that fluid therapy and resultant diuresis prevented such derangements from manifesting in this case.

Development of acute kidney injury associated with rhabdomyolysis has been described in the veterinary literature [16–19]. The mechanism of kidney injury proposed in rhabdomyolysis is multifactorial and includes some combination of renal hypoperfusion, ischemic damage, free radical formation, and acute tubular necrosis [8, 9, 18]. In this patient, specific treatment for rhabdomyolysis included only fluid diuresis in accordance with the most commonly recommended clinical approach [4, 20, 21]. Ultimately, acute kidney injury was not apparent during the course of treatment in this case based upon serial blood chemistries. A limitation of our case is that a routine urinalysis was not performed on the sample acquired at presentation due to a limited sample volume. With ongoing fluid diuresis and myoglobinuria, it was determined that any urine sample taken during the course of hospitalization would be of limited utility for assessing renal status. On post hoc consideration, a urine sediment would have, at minimum, allowed for assessment of cast formation.

Amphetamines are known to enhance serotonin release and can result in clinical signs relating to serotonin syndrome [4]. Paradoxical reactions to benzodiazepines are known to occur in amphetamine toxicity and are suggested to be caused by disinhibition of serotonin-derived behaviors [4, 22]. This may explain both the exacerbation of hyperactivity after midazolam administration and the effectiveness of cyproheptadine in this patient. The patient's response to sedative treatment was important in narrowing the differentials and arriving at a tentative diagnosis of amphetamine toxicity in this patient.

The inclusion of antioxidant therapy in this case may have provided an additional benefit as suggested by other reports of severe rhabdomyolysis in dogs [19, 23]. The free radical scavenger effects cited in these cases, however, were a product of the mechanistic spectrums of lidocaine and mannitol use rather than the S-adenosylmethionine administered in this patient. Further study is necessary to establish whether a clinical benefit exists with the use of antioxidants in canine rhabdomyolysis or as a preventative measure against the acute kidney injury that may be associated with it.

In conclusion, this report describes a case of severe rhabdomyolysis associated with amphetamine toxicity in a dog. Despite the severity of the rhabdomyolysis in this case, acute kidney injury may have been avoided with supportive therapies alone. Development of rhabdomyolysis in dogs with amphetamine toxicity continues to be considered an uncommon occurrence, but the true prevalence will require further study.

## Figures and Tables

**Table 1:** Serial serum chemistries from initial presentation and throughout treatment.

Parameter	Units	Result (initial)	Result (day 2)	Result (day 3)	Result (day 5)	Result (day 9)	Reference range
GLU	mmol/L	9.7	5.5	8	7.4	6.3	4.4-6.4
AST	U/L	176	4,251	3,368	511	34	0-50
ALT	U/L	27	342	526	565	127	0-60
ALP	U/L	25	127	156	122	56	0-100
CK	U/L	1,481	160,610	181,900	4,410	292	0-200
TBIL	*μ*mol/L	41	3.4	5.1	<1.7	1.7	0-6.8
TP	g/L	73	60	55	56	63	58-75
ALB	g/L	32	28	25	25	26	26-42
GLOB	g/L	41	32	30	31	37	25-40
BUN	mmol/L	9.6	5.7	7.1	3.9	5.4	2.9-7.9
CREA	*μ*mol/L	132.6	103.4	83.1	61.9	76.9	44.2-150.3
CA	mmol/L	2.2	2.53	2.25	2.17	2.48	2.45-2.85
PHOS	mmol/L	0.81	1.74	1.07	1.07	1.45	1.10-2.03
NA	mmol/L	161	160	144	154	144	140-153
K	mmol/L	4.5	3.2	3.8	4.2	3.7	3.8-5.5
CL	mmol/L	124	126	114	117	111	107-115

PCV: packed cell volume; GLU: glucose; AST: aspartate aminotransferase; ALT: alanine aminotransferase; ALP: alkaline phosphatase; CK: creatine kinase; TBIL: total bilirubin; TP: total protein; ALB: albumin; GLOB: globulins; BUN: blood urea nitrogen; CREA: creatinine; CA: calcium; PHOS: phosphorous; NA: sodium; K: potassium; and CL: chloride.
